# Draft Genome Sequences of Acinetobacter harbinensis Strain HITLi 7^T^, Isolated from River Water

**DOI:** 10.1128/genomeA.00028-15

**Published:** 2015-03-05

**Authors:** Duoying Zhang, Weiguang Li, Wen Qin, Xiaofei Huang, Xujin Gong

**Affiliations:** a School of Municipal and Environmental Engineering, Harbin Institute of Technology, Harbin, Heilongjiang, China; b State Key Laboratory of Urban Water Resource and Environment, Harbin, Heilongjiang, China

## Abstract

Acinetobacter harbinensis HITLi strain 7^T^, isolated from river water, has the ability to remove ammonium and organic chemicals at 2°C. The genome sequences might be useful for investigating the low-temperature adaptability and nitrogen or organic chemical metabolism.

## GENOME ANNOUNCEMENT

Acinetobacter harbinensis HITLi strain 7^T^ (KCTC 32411) was isolated from the Songhua River near Harbin, China. Recently, it was classified as a novel species of the genus Acinetobacter (1). A. harbinensis HITLi 7^T^ showed ability of removing ammonium and organic chemicals at 2°C. Study on the genome sequence of A. harbinensis HITLi 7^T^ might provide useful insight into its characteristics of cold adaptability and nitrogen or organic chemical metabolism.

The genome of A. harbinensis HITLi 7^T^ was sequenced using the Illumina HiSeq2000 platform; 452 Mb of data were produced, and 5,421,704 reads were assembled using SOAP *de novo* version 2.04 (2, 3). The assembled genome has a length of 2,855,331 bp and consists of 160 contigs (*N*_50_, 41,669 bp). The overall G+C content was 41.54%. The gene prediction was performed using Glimmer version 3.02 (http://ccb.jhu.edu/software/glimmer/index.shtml) (4). The tandem repeats were predicted using the Tandem Repeats Finder (5). Functional annotation was completed by BLAST comparison to the NCBI nonredundant public database. One sRNA, 76 tRNAs, 2 copies of 5S rRNA, and 3 copies each of 16S rRNA and 23S rRNA were identified using RNAmmer (6).

The wax ester synthase/acyl-CoA:diacylglycerol acyltransferase (*Wsd*1) gene was present in the genome of A. harbinensis HITLi 7^T^, which was the important factor for cold adaptation (7). A series of autotrophic carbon dioxide fixation genes were found, such as acetyl-CoA carboxylase, pyruvate synthase, pyruvate:water dikinase, and PEP carboxylase. Among the set of genes, 183 genes were associated with xenobiotic biodegradation.

### Nucleotide sequence accession numbers.

This whole-genome shotgun project has been deposited at DDBJ/EMBL/GenBank under the accession number JXBK00000000. The version described in this paper is version JXBK01000000.
